# Comparative Analysis the Complete Chloroplast Genomes of Nine *Musa* Species: Genomic Features, Comparative Analysis, and Phylogenetic Implications

**DOI:** 10.3389/fpls.2022.832884

**Published:** 2022-02-10

**Authors:** Weicai Song, Chuxuan Ji, Zimeng Chen, Haohong Cai, Xiaomeng Wu, Chao Shi, Shuo Wang

**Affiliations:** ^1^College of Marine Science and Biological Engineering, Qingdao University of Science and Technology, Qingdao, China; ^2^Department of Life Sciences, Imperial College London, Silwood Park, London, United Kingdom; ^3^Plant Germplasm and Genomics Center, Germplasm Bank of Wild Species in Southwest China, Kunming Institute of Botany, Chinese Academy of Sciences, Kunming, China

**Keywords:** *Musa*, chloroplast genome, genetic structure, comparative analysis, phylogenetic analysis, interspecific relationships

## Abstract

*Musa* (family Musaceae) is monocotyledonous plants in order Zingiberales, which grows in tropical and subtropical regions. It is one of the most important tropical fruit trees in the world. Herein, we used next-generation sequencing technology to assemble and perform in-depth analysis of the chloroplast genome of nine new *Musa* plants for the first time, including genome structure, GC content, repeat structure, codon usage, nucleotide diversity and etc. The entire length of the *Musa* chloroplast genome ranged from 167,975 to 172,653 bp, including 113 distinct genes comprising 79 protein-coding genes, 30 transfer RNA (tRNA) genes and four ribosomal RNA (rRNA) genes. In comparative analysis, we found that the contraction and expansion of the inverted repeat (IR) regions resulted in the doubling of the *rps19* gene. The several non-coding sites (*psbI–atpA, atpH–atpI, rpoB–petN, psbM–psbD, ndhf–rpl32*, and *ndhG–ndhI*) and three genes (*ycf1, ycf2*, and *accD*) showed significant variation, indicating that they have the potential of molecular markers. Phylogenetic analysis based on the complete chloroplast genome and coding sequences of 77 protein-coding genes confirmed that *Musa* can be mainly divided into two groups. These genomic sequences provide molecular foundation for the development and utilization of *Musa* plants resources. This result may contribute to the understanding of the evolution pattern, phylogenetic relationships as well as classification of *Musa* plants.

## Introduction

Musaceae is a small family of Zingiberales in monocotyledonous plants, mostly distributed in tropical regions in Australia, Africa, and Asia. It is closest to Strelitziaceae, Lowiaceae, and Heliconiaceae in phylogenetic position (Kress et al., 2001). Three genera are commonly recognized within Musaceae. *Ensete* is a small genus with eight to nine species found in Madagascar, sub-Saharan Africa and Asia, *Musella* is a monotypic genus native to southwest China (Li et al., 2010). While most species of the family, which occur mainly in Southeast Asia, are classified into the *Musa* group (Häkkinen and Väre, 2008). *Musa* grow in tropical and subtropical regions and is one of the most important tropical fruit trees in the world. According to molecular analysis, wild *Musa* species are reclassified into two groups, *Musa* L. sect. *Musa* (by merging *Eumusa* with *Rhodochlamys*) and *Musa* sect. *Callimusa*, including the previously classified *M*. sect. *Australimusa* and *M*. sect. *Ingentimusa* (Häkkinen, 2013). Banana fiber has become one of the high potential biological resources in new material field due to it’ s characteristics such as sustainability, low cost and environmental friendliness (Pappu et al., 2015; Vishnuvarthanan et al., 2019). For example, the leaf fibers of abaca (*Musa textilis*) are ideal raw materials for manufacturing specialty paper (del Río and Gutiérrez, 2006). Many organs of *Musa* plants are being used in various fields. Banana peels not only have effect in purifying Cr(III), Cr(VI), Cu(II), and radioactive substances (uranium and thorium) in water (Pakshirajan et al., 2013; Oyewo et al., 2016), but also were used as a new type of bio-sorbent to adsorb aflatoxins and ochratoxin A (Shar et al., 2016). Tree trunks and leaves can be used as precursors for the production of adsorbents for the purification of various pollutants (Ahmad and Danish, 2018). The dry biomass of banana pseudo stem can remove the reactive blue 5G (RB5G) dye (Jarvis and López-Juez, 2013). At the same time, many parts of banana can be used to produce industrial raw materials, such as ethanol, polyhydroxy butyrate (PHB), etc. (Oberoi et al., 2011; Ingale et al., 2014; Naranjo et al., 2014). Banana starch also plays an important role in the food, pharmaceutical, and cosmetic industries (Ramírez-Hernández et al., 2017; Arias et al., 2021; Taweechat et al., 2021; Thanyapanich et al., 2021).

Chloroplasts are an energy converter that provides energy for higher plants and algae, which are a unique structure of plant cells. At the same time, chloroplasts play a vital role in many functions of plant growth, including starch storage, sugar synthesis, the production of several amino acids, lipids, vitamins and pigments, essential sulfur and nitrogen metabolic pathways (Jarvis and López-Juez, 2013; Martin et al., 2013; Nielsen et al., 2016). In angiosperms, chloroplast (cp) genome is mainly a circular structure with the length is between 120–180 kb (Provan et al., 2001). The chloroplast genome is a circular double-stranded structure, which is divided into four parts, two of which are called single-copy regions, including a large single-copy region (LSC) and a small single-copy region (SSC) (Kolodner and Tewari, 1979), and the other two almost identical regions separating the single-copy regions are called inverted repeat sequences A and B (IRa, IRb) (Wicke et al., 2011). Compared with the nuclear and mitochondrial genomes, the chloroplast genome is relatively conserved in gene structure and composition (Asaf et al., 2017a). With the rapid development of Next Generation Sequencing (NGS), the National Center for Biotechnology Information (NCBI) database provides more and more chloroplast genomes, enabling people to have a better understanding of the relationship between chloroplast structure and genetic evolution, which also heavily facilitated the research of chloroplast genomes (Yang et al., 2014; Li et al., 2017; Amiryousefi et al., 2018b). The polymorphic sites of the chloroplast genome can be used to develop reliable and stable molecular markers, which will help us to study population genetics and phylogeny (Ahmed et al., 2013; Sheng et al., 2021).

The relatively conservative chloroplast genome is an ideal research method for studying genetic relationship identification. It is of great significance to analyze the chloroplast genome of *Musa*, including structural characteristics, phylogenetic relationships and population genetics. As a supplementary technology, chloroplast sequencing not only provide part of the genetic diversity information about *Musa* germplasm resources, but also clarifies the genes and potential functions of *Musa* plants. So far, the complete chloroplast sequences of *Musa* plants have been obtained in *Musa acuminata* (Martin et al., 2013), *Musa balbisiana* (Shetty et al., 2016), *Musa beccarii* (Feng et al., 2020) and *Musa ornate* (Liu et al., 2018) and so forth. Here, we reported the complete chloroplast genomes of nine *Musa* species, which was the first comprehensive comparison of these nine species. We compared the structure and content patterns of nine *Musa* chloroplast genomes; explored the sequence differences in nine *Musa* cp genomes; detected simple sequence repeats (SSR) and long repeats; calculated codon usage bias and putative RNA editing site. We also studied the genetic variation between *Musa* species, including inverted repeat (IR) contraction/expansion; gene duplication and loss during evolution; the ratio of non-synonymous (*K*_*a*_) to synonymous substitutions (*K*_*s*_), which may help uncover the genetic relationship between *Musa* species. We also performed phylogenetic analyses using chloroplast genome sequences from other related species to further determine the taxonomy of *Musa* genus. These results perfect the existing genetic information of *Musa* species and provide a valuable reference for the DNA molecular research of *Musa* species. Application of these results will help assess the genetic variation and phylogenetic relationships between closely related species and support the development of wild germplasm resources.

## Materials and Methods

### Sample Collection, DNA Extraction, and Sequencing Plants

In this study, the nine species of *Musa* were collected from Plant Germplasm and Genomics Center, Kunming Institute of Botany, the Chinese Academy of Sciences, and was approved by Kunming Institute of Botany and local policy. The voucher specimen and DNA were deposited at Qingdao University of Science and Technology (specimen code BJ210253-BJ210261). Total genomic DNA was extracted from fresh leaves using modified CTAB (Porebski et al., 1997). According to the manufacturer’s protocol, the Illumina TruSeq Library Preparation Kit (Illumina, San Diego, CA, United States) was used to prepare approximately 500 bp of paired-end libraries for DNA inserts. These libraries were sequenced on the Illumina HiSeq 4000 platform in Novogene (Beijing, China), generating raw data of 150 bp paired-end reads. About 3 Gb high quality, 2 × 150 bp pair-end raw reads were obtained and were used to assemble the complete chloroplast genome of *Musa.*

### Chloroplast Genome *de novo* Assembly and Annotation

Trimmomatic 0.39 software were used preprocessed the raw data (Bolger et al., 2014), including removal of adapter sequences and other sequences introduced in the sequencing, removing low-quality and over-N-base reads, etc. The quality of newly produced clean short reads was assessed using FASTQC v0.11.9 and MULTIQC software (Ewels et al., 2016), and high-quality data with Phred scores averaging above 35 were screened out. According to the reference sequence (*Musa balbisiana*), the chloroplast-like (cp) reads were isolated from clean reads by BLAST (Shetty et al., 2016). Short reads were *de novo* assembled into long contigs using SOAPdenovo 2.04 (Luo et al., 2012) by setting kmer values of 35, 44, 71, and 101. Furthermore, the long-contigs was expanded and gap-filed using Geneious ver 8.1 (Muraguri et al., 2020), which forms the whole chloroplast genome. The complete chloroplast genome was further validated and calibrated by using *de novo* splicing script NOVOplsty 4.2 (Dierckxsens et al., 2017). In addition, GeSeq (Tillich et al., 2017) was used to annotate the *de novo* assembled genomes, RNAmmer (Lagesen et al., 2007) was used to validate rRNA genes with default settings, and tRNAscanSE ver 1.21 (Lowe and Eddy, 1997) was applied to detect tRNA genes with default settings. Finally, we compared the results with the reference sequence and corrected the misannotated genes by GB2Sequin (Lehwark and Greiner, 2019) in an artificial way. The circular map of the genomes was drawn by using Organellar Genome DRAW (OGDRAW) (Lohse et al., 2007). The nine newly assembled *Musa* chloroplasts genomes were deposited in GenBank with the accession numbers NC_056826 - NC_056834.

### Plastome Structural Analysis

Chloroplast Microsatellites or simple sequence repeats (SSRs) were detected in the perl script MISA (Beier et al., 2017). The basic repeat setting of SSRs was determined: ten for mononucleotide, five for dinucleotide, four for trinucleotide and three for tetranucleotide pentanucleotide hexanucleotide. The REPuter tool (Kurtz et al., 2001) was applied to analyze forward (F), reverse (R), complement (C), and palindromic (P) oligonucleotide repeats. The following parameters were used to identify repeats with: (1) hamming distance equal to 3; (2) minimal repeat size set to 30 bp; and (3) maximum computed repeats set to 300 bp. Relative synonymous codon usage (RSCU) and amino acid frequency in the protein coding gene region were determined by MEGA-X (Kumar et al., 2018). The putative RNA editing sites in 35 genes were investigated in the coding gene using PREP-cp (Predictive RNA Editors for Plants chloroplast) (Mower, 2009).

### Genome Comparison

We compared and analyzed the basic features of nine chloroplast genomes using Geneious software, including calculating the length of the region sequence, GC content in different regions, and the proportions of different sequences. The junction sites of various regions of the chloroplast genome were analyzed in IRscope (Amiryousefi et al., 2018a) to visualize the expansion and contraction of reverse repeats (IR). We used KaKs_Calculator 2.0 software (Wang et al., 2010) to calculate the rate values of *K*_*s*_ (synonymous substitution) and *K*_*a*_ (non-synonymous substitution) with the YN method. Shuffle-LAGAN mode alignment program in mVISTA (Brudno et al., 2003) was used to evaluate structural similarity for the nine species, with the annotation of *M. balbisiana* as the reference.

### Phylogenetic Analysis

The complete chloroplast genomic sequences from 17 species of *Musa* (nine sequences newly generated and eight species obtained from GenBank) were performed for phylogenetic analyses (Supplementary Table 8). *Heliconia collinsiana* (accession number NC_020362) and *Ravenala madagascariensis* (accession number NC_022927) were downloaded from the NCBI (National Center of Biotechnology Information) as an outgroup of the evolutionary tree. Multiple sequence alignment was aligned using MAFFT and GTR-GAMMA (GTR + G) model was selected using model test applying the Bayesian information criterion (BIC) (Posada and Crandall, 1998). All InDels were excluded from the alignment sequence to construct a phylogenetic tree based on only substitutions. The maximum likelihood (ML) trees were conducted by MEGA-X and 1,000 bootstrap replicates were set to evaluate the branch support values. Finally, the 79 protein-coding genes from the 19 species were also extracted to reconstructed ML trees using the same methods.

## Results

### Assembly and Annotation of the Chloroplast Genomes of Nine *Musa* Species

Genome-skimming data were generated about 3.2–5.7 GB by the Illumina HiSeq 2500 in each of the sequenced *Musa* species. The complete chloroplast genomes of these nine species were typical circular double-stranded structures and ranged from 167,975 bp (*Musa jackeyi*) to 172,653 bp (*Musa rubinea*) (Table 1). All nine sequence presented the quadripartite structure, including large single copy (LSC) region, the small single copy (SSC) region and a pair of inverted repeat (IR) regions. The length of the LSC region ranged between 88,330 and 89,997 bp, with the GC content of 34.8–35.2%. The length of the SSC region was distributed between 10,773 and 11768 bp. The GC content of SSC regions was similar in nine species, ranging from 30.1% in *M. rubinea* to 31.2% in *Musa laterita*. 33,864–35,522 bp was the length range of the IR region of nine *Musa* species, which contains 39.5–40.0% GC content. The complete chloroplast genome sequences of the nine *Musa* species were provided in GenBank (under accession number NC_056826–NC_056834).

**TABLE 1:** Chloroplast genome features of nine species of *Musa*.

Genome features	*Musa ingens*	*Musa jackeyi*	*Musa laterita*	*Musa lolodensis*	*Musa mannii*	*Musa nagensium*	*Musa rubinea*	*Musa troglodytarum*	*Musa yunnanensis*
Genome size (bp)	168,471	167,975	170,565	168,542	170,699	170,304	172,653	168,121	169,816
LSC size (bp)	89,888	88,422	88,748	88,330	88,883	88,420	89,997	88,724	90,720
SSC size (bp)	10,855	11,049	10,773	11,060	10,816	11,082	11,768	11,049	11,072
IR size (bp)	33,864	34,252	35,522	34,576	35,500	35,401	35,444	34,174	34,012
Total GC content (%)	36.8	36.9	36.8	36.8	36.8	36.6	36.4	36.8	36.8
GC content in LSC (%)	35.2	35.1	35.2	35.2	35.1	35.0	34.8	35.1	35.1
GC content in SSC (%)	31.0	31.1	31.2	31.1	31.2	30.8	30.1	31.1	31.1
GC content in IR (%)	39.6	40.0	39.5	39.9	39.5	39.5	39.5	40.0	39.9
Number of genes (unique)	135(113)	135(113)	136(113)	135(113)	136(113)	136(113)	136(113)	135(113)	136(113)
Protein-coding genes (unique)	89(79)	89(79)	90(79)	89(79)	90(79)	90(79)	90(79)	89(79)	90(79)
tRNA genes (unique)	38(30)	38(30)	38(30)	38(30)	38(30)	38(30)	38(30)	38(30)	38(30)
rRNA genes (unique)	8(4)	8(4)	8(4)	8(4)	8(4)	8(4)	8(4)	8(4)	8(4)
Accession numbers in GenBank	NC_056826	NC_056827	NC_056828	NC_056829	NC_056830	NC_056831	NC_056832	NC_056833	NC_056834

**FIGURE 1:**
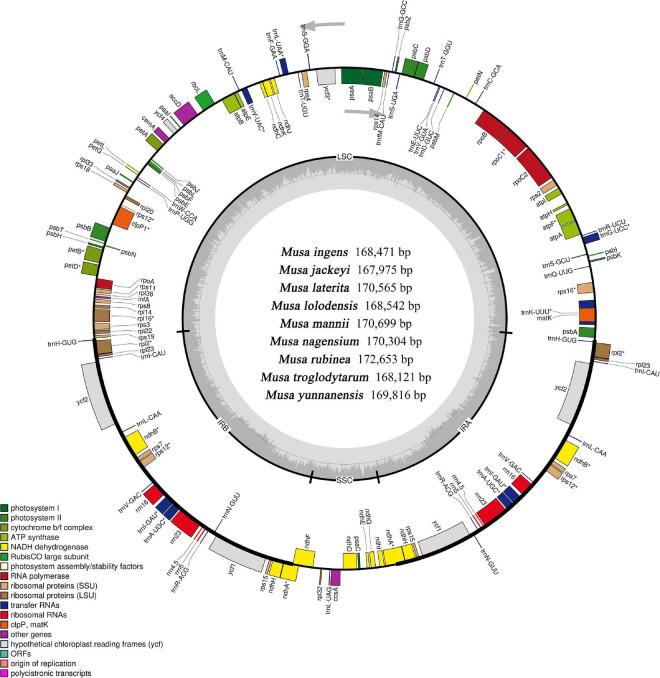
Complete genome map of the chloroplast genome of *Musa*. The inner gray ring is divided into four areas, Clockwise, and they are: small single-copy region (SSC), inverted repeat sequences B (IRb), large single-copy region (LSC), and inverted repeat sequences A (Ira). The genes in the outer ring region are transcribed clockwise, while those in the inner ring are transcribed counterclockwise. The inner ring dark gray indicates the GC content, the light gray reaction AT content. In the lower left is a legend that classifies chloroplast genes according to their functions.

Although the length of the chloroplast genomes of the nine species was some different, the analyses of the genetic composition showed that they have some similarities. The positions of the genes were visualized in Figure 1. A total of 135 functional genes were predicted in all nine *Musa.* sps, including 113 unique genes comprising 79 protein-coding genes, 30 transfer RNA (tRNA) genes and four ribosomal RNA (rRNA) genes. These genes, represented by *Musa nagensium*, can be roughly divided into three categories: photosynthesis-related genes, chloroplast self-replication genes, and other genes (Table 2). Among the genes, 18 intron-containing genes (ICG) were found, covering 12 protein-coding genes and 6 tRNA genes (Supplementary Table 1). Among these ICG, *ycf3*, and *clpP* possessed two introns, respectively, while the rest of ICG contained only one intron. The *rps12* gene has *trans*-splicing, and its 3′-end is duplicated in the IRs region, while its 5′-end is present in the LSC region. As a regional demarcation gene, the *ndhA* gene starts at the IRs region and ends at the SSC region.

**TABLE 2:** List of predicted genes in the *Musa* chloroplast genome.

Category for gene	Group of gene	Name of gene				
	Subunits of Photosystem I	*psaA*	*psaB*	*psaC*	*psaI*	*psaJ*
	Subunits of Photosystem II	*psbA*	*psbB*	*psbC*	*psbD*	*psbE*
		*psbF*	*psbH*	*psbI*	*psbJ*	*psbK*
		*psbL*	*psbM*	*psbN*	*psbT*	*psbZ*
Genes for photosynthesis	Subunits of NADH oxidoreductase	*ndhA* ^(ab)^	*ndhB* ^(ab)^	*ndhC*	*ndhD*	*ndhE*
		*ndhF*	*ndhG*	*ndhH* ^(a)^	*ndhI*	*ndhJ*
		*ndhK*				
	Cytochrome b6/f complex	*petA*	*petB*	*petD*	*petG*	*petL*
		*petN*				
	Subunits of ATP synthase	*atpA*	*atpB*	*atpE*	*atpF* ^(b)^	
		*atpI*				
	Large subunit of RuBisCo	*rbcL*				
	Large subunit of ribosomal proteins	*rps2*	*rps3*	*rps4*	*rps7* ^(a)^	*rps8*
		*rps11*	*rps12* ^(ab)^	*rps14*	*rps15* ^(a)^	*rps16* ^(b)^
		*rps18*	*rps19* ^(a)^			
	Small subunit of ribosomal proteins	*rpl2* ^(ab)^	*rpl14*	*rpl16*	*rpl20*	*rpl22*
		*rpl23* ^(a)^	*rpl32*	*rpl33*	*rpl36*	
	DNA-dependent RNA polymerase rRNA	*rpoA*	*rpoB*	*rpoC1* ^(b)^	*rpoC2*	
Self-replication	Ribosomal RNA genes	*rrn4.5^(a)^*	*rrn5* ^(a)^	*rrn16* ^(a)^	*rrn23* ^(a)^	
	Transfer RNA genes	*trnA-UGC^(ab)^*	*trnC-GCA*	*trnD-GUC*	*trnE-UUC*	*trnF-GAA*
		*trnfM-CAU*	*trnG-GCC^(ab)^*	*trnH-GUG^(a)^*	*trnI-CAU^(a)^*	*trnI-GAU^(ab)^*
		*trnK-UUU^(b)^*	*trnL-CAA^(a)^*	*trnL-UA^(ab)^*	*trnL-UAG*	*trnM-CAU*
		*trnN-GUU^(a)^*	*trnP-UGG*	*trnQ-UUG*	*trnR-ACG^(a)^*	*trnR-UCU*
		*trnS-GCU*	*trnS-GGA*	*trnS-UGA*	*trnT-GGU*	*trnT-UGU*
		*trnV-GAC^(a)^*	*trnV-UAC^(b)^*	*trnW-CCA*	*trnY-GUA*	
	Translational initiation factor	*infA*				
Other genes	Maturase	*matK*				
	Protease	*clpP* ^(c)^				
	Envelope membrane protein	*cemA*				
	Subunit acetyl-CoA-carboxylase	*accD*				
	c-Type cytochrome synthesis gene	*ccsA*				
	Conserved open reading frames	*ycf1* ^(a)^	*ycf2* ^(a)^	*ycf3* ^(c)^	*ycf4*	

*^(a)^Two gene copies in IRs; ^(b)^gene containing a single intron; ^(c)^gene containing two introns.*

### Codon Usage Bias

In this study, we analyzed the codon usage bias and relative synonymous codon usage (RSCU) based on the protein coding gene of *Musa*’s chloroplast genome, and a total of 28,690–29,360 codons were identified (Supplementary Table 2). Analysis showed that codons containing A or T instead of C or G at the 3′-end of the codon have a higher encoding rate. The RSCU of codons containing A/T at the 3′-end was mainly greater than 1, and the codons containing C or G at the 3′-end mostly have RSCU ≤ 1. In addition, there were 29 codons with RSCU values greater than 1, 2 of them were equal to 1, and 30 of them are less than 1. Among them, AUU (4.15–4.24%, Isoleucine), AAA (4.15–4.36%, Lysine), and GAA (4.21–4.39%, Glutamic acid) were the most frequently used codons, while UGC (0.30–0.31%, Cysteine) and CGC (0.31–0.33%, Arginine) had the lowest usage rates. In addition, most amino acids possessed at least two synonymous codons, except for methionine (AUG) and tryptophan (UGG), which had no codon usage preference since they only have one coding codon. Among all codons with an RSCU value greater than 1, the vast majority of codons presented a higher A/T appreciation in the third codon. Overall, we found that the nine *Musa* species have high similarities in codon usage and amino acid frequency. This result is very common in the chloroplast genome of higher plants (Gichira et al., 2017).

### Positive Selection Analysis and Putative RNA Editing Site

The ratio of non-synonymous (*K*_*a*_) to synonymous substitutions (*K*_*s*_), *K*_*a*_/*K*_*s*_, has been widely used to evaluate the natural selection pressure and evolution rates of nucleotides in genes (Li et al., 1985). The results of the statistical neutrality test indicated that 77 protein-coding genes were relatively stable during the evolution process, but two genes (*ycf1* and *ycf2*) were under positive selection (Supplementary Table 3). The *K*_*a*_/*K*_*s*_ ratio of the *ycf2* gene of the nine species in *Musa* are all greater than 1 (2.66–5.22). Except for the *K*_*a*_/*K*_*s*_ ratio of the *ycf1* gene of *Musa mannii* (0.9), that of the other eight species are also all positive selection status (1.16–2.29).

In order to gain a deeper insight into the RNA metabolism of *Musa* species, we used PREP to predict 74–77 post-transcriptional RNA editing modifications of 26 protein-coding genes (Supplementary Table 4). Most RNA editing sites were located in *ndhB* (11 editing sites, 14.3–14.8%), while *ndhD* (5–7 editing sites, 6.6–9.2%), *ndhF* (6–7 editing sites, 7.8–9.4%), and *rpoB* (5–6 editing sites, 6.7–8.0%) also had a great portion of editing sites. The types of RNA editing sites reported here were all C to U and all affect a single site. All changes occurred in the first or second nucleotides of the codon. Among the amino acid conversions caused by RNA editing sites, the transformation of serine to leucine accounted for one-third of the total.

### Inverted Repeat Contraction and Expansion

**FIGURE 2:**
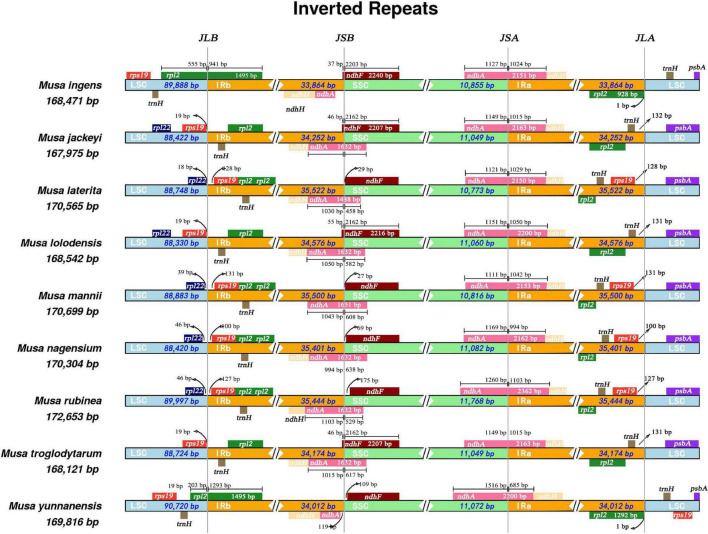
Comparison of SSC, LSC, IRB, and IRB boundary regions in the chloroplast genomes of nine species of *Musa*. Comparative analysis of junction sites in *Musa* chloroplast genomes. The coordinate position of the start or end of each gene from the corresponding junction is shown with arrows. All the genes those integrate from one region of chloroplast genome to another region are shown with the T bar above or below. The T bars show the length of base pair for which the integration of genes has been occur. JLA (IRa/LSC), JLB (IRb/LSC), JSA (SSC/IRa), and JSB (IRb/SSC) denote the junction sites between each corresponding two regions on the genome.

Our research revealed that all nine *Musa* species have *ndhA* genes that spanned the SSC and IRa regions (Figure 2). Only the *ndhA* gene of *Musa yunnanensis* was longer in the SSC region than in the IRa region. In comparison, the length of the *ndhA* gene of the remaining eight species were not much different in the SSC and IRa regions. We speculated that this may be due to the expansion of the IRa region of *M. yunnanensis*. Primarily, at the junction of LSC/IRb (JLB), the *rpl2* gene was located in the IRb region, while the *rpl2* genes of *Musa ingens* and *M. yunnanensis* spanned the LSC and the IRb region. According to the distribution of *rps19* gene, nine *Musa* species can be roughly divided into three categories. The *rps19* gene of *M. laterita, M. mannii, M. nagensium*, and *M. rubinea* in the first category were entirely located in the IR region, 100–131 bp apart from LSC/IRb and IRa/LSC. The second type of species (*M. jackeyi, Musa lolodensis*, and *Musa troglodytarum*) were where the *rps19* gene was situated at the junction of LSC/IRb and were 18–19 bp away from the IRb region. Moreover, the *rps19* genes were entirely located in the IRb region (*M. ingens and M. yunnanensis*), suggesting that this phenomenon may occur with the contraction IRb area. However, at the junction of IRa/LSC (JLA), *M. yunnanensis* processed two *rps19* genes, so we speculated that *rps19* was deleted in *M. ingens*.

### Repeat Sequence and Simple Sequence Repeats Analysis (Analysis of Microsatellites and Oligonucleotide Repeats)

**FIGURE 3:**
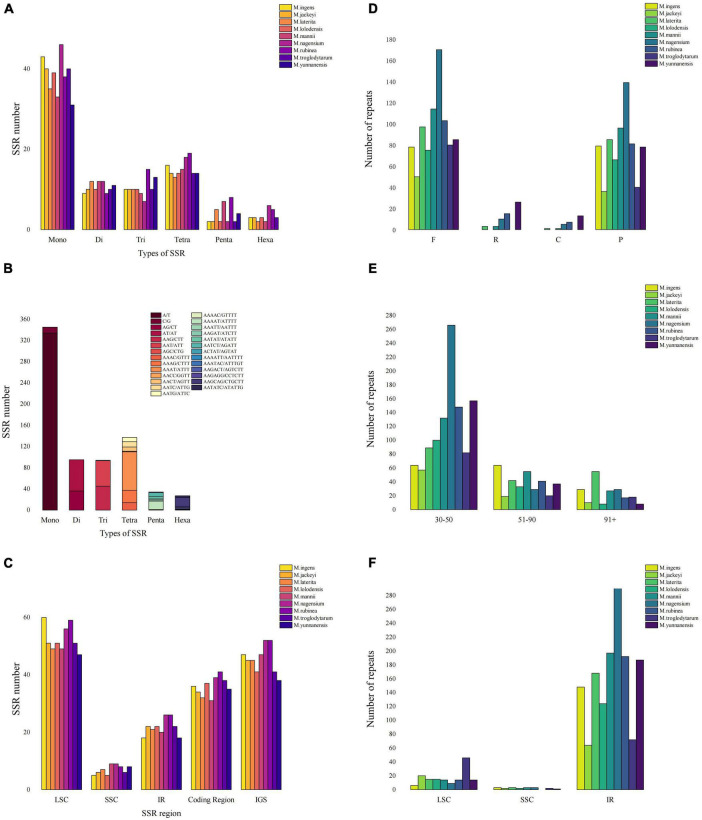
Comparison of microsatellites and long repeats in the chloroplast genomes of *Musa* species. (A) The number of SSRs of different types of SSR for nine *Musa* species. (B) Details in SSR types among nine *Musa* species. (C) The number of SSR markers in the LSC/SSC/IR region along with coding region and IGS. (D) Number of four long repeat sequences in nine species: complement repeats. F represents forward repeats, P represents palindromic repeats, R represents reverse repeats, C represents complement repeats. (E) Number of long repeat sequences with different lengths in nine species. (F) The distribution of long repeats in LSC, SSC and IR regions.

This study counted all the interspersed repetitive sequences in the *Musa* chloroplast genome with a repeat unit length of more than 30 bp. At the same time, we detected four types of repeats, including forward repeats (F), inverted repeats (R), complementary repeats (C), and palindromic repeats (P) (Supplementary Table 5). Repeat analysis showed 50–170 forward duplications, 0–26 inverted duplications, 0–13 complementary repeats and 37–140 palindromic repeats in nine *Musa* species (Figure 3A). The length of the repetitive sequence varied from species to species, but most of the repetitive sequence length existed in the range of 30–50 bp (40.76–82.1%) (Figure 3B). Compared with the LSC and SSC regions, the IR region contained most of the repetitive sequences, and the chloroplast genome regions also shared most of the repetitive sequences. Among them, the repetitive sequences in the IR region of *M. nagensium* accounted for the highest proportion of all repetitive sequences (96.3%), and the IR region of *M. troglodytarum* had the lowest proportion of repetitive sequences (60%) (Figure 3C).

We analyzed the simple sequence repeats (SSRs) in the chloroplast genomes of nine *Musa* species (Figure 3D). A total of six types of SSR (mono-/di-/tri-/tetra-/penta-/hexa-nucleotide repeats) were detected, the first four microsatellites accounted for 86.17–94.52%, and the penta- or hexanucleotide repeats was very small (no more than 8) or even non-existent (Figure 3E). In the MISA analysis, the number of SSRs detected in the nine *Musa* species was 73–93. At the same time, the distribution of SSR in the LSC region (61.54–72.29%) was higher than that in the IR region (21.69–28.57%) and SSC region (6.02–11.54%) (Figure 3F). Analysis revealed that SSRs were mainly distributed in the non-coding areas (51.9–60.26%). The number of SSRs in the coding region of *M. rubinea* (41) were the largest, while that of *M. mannii* (31) was the lowest.

### Sequence Divergence in the Nine *Musa* Chloroplast Genomes

Using *M. Balbisiana* as a reference, we used the DnaSP6 to detect single nucleotide polymorphisms (SNPs) in the chloroplast genomes of nine *Musa* species (Table 3). Through analysis, we divided these nine species into two groups. The first group contained four species (*M. ingens, M. jackeyi, M. lolodensis*, and *M. troglodytarum*). In comparison, the second group comprised five species (*M. laterita, M. mannii, M. nagensium, M. rubinea*, and *M. yunnanensis*). Among them, 1,419–1,459 SNPs were detected in the first group of four species, and 628–716 SNP sites were seen in the second group. We found that the distribution of SNPs of the nine species in the LSC region is not much different, and the SNP of each species accounted for the highest proportion in the LSC region (61.17–65.29%) (Supplementary Table 6). However, in the statistics of SNP content in the IR region, the ratio of the first group (16.30–19.97%) was slightly lower than that of the second group (21.85–22.62%), and the proportion of SNP in the SSC region was slightly higher (the first group: 15.45–21.04%, the second group: 14.16–14.59%). The mutation frequencies of the corresponding LSC, SSC, and IR regions of the first group were 1.020–1.035, 1.846–1.953, and 0.448–0.487%, respectively, while the second group was 0.452–0.497, 0.876–1.281, and 0.155–0.202%. We also analyzed the insertions and deletions of the chloroplast genomes of nine species, which found they have similar rules in SNPs. 126–160 insertions were detected in nine species, respectively. The detection rates of LSC, SSC and IR were 61.90–67.42, 3.79–14.81, and 20.99–30.60%. The deletion mainly occurred in the LSC region (the first group: 58.66–66.64%, the second group: 59.09–66.04%), followed by the IR region (the first group: 26.06–30.60%, the second group 16.39–24.82%), and finally, the SSC region (the first group: 9.84–15.08%, the second group: 13.21–22.13%).

**TABLE 3:** Details of single nucleotide polymorphisms (SNP) and InDel sites in large single-copy region (LSC), small single-copy region (SSC), and inverted repeat (IR) regions in the complete chloroplast genomes of nine *Musa* species.

SNP and indel			*M. ingens*	*M. jackeyi*	*M. laterita*	*M. lolodensis*	*M. mannii*	*M. nagensium*	*M. rubinea*	*M. troglodytarum*	*M. yunnanensis*
Number	SNP	Transition	954	917	361	912	349	322	365	915	347
		Transversion	505	524	309	507	342	353	351	528	281
		Total	1459	1441	670	1419	691	675	716	1443	628
	Insertion		160	132	134	155	143	139	162	132	126
	Deletion		179	180	122	165	132	88	106	183	141
Region	SNP	LSC	917	915	423	902	442	423	438	914	410
		SSC	212	204	114	207	116	142	135	205	97
		IR	330	322	133	310	133	110	143	324	121
	Insertion	LSC	103	87	84	96	94	90	104	89	78
		SSC	14	5	9	14	12	15	24	14	12
		IR	43	40	41	45	37	34	34	29	36
	Deletion	LSC	105	109	75	105	78	56	70	109	86
		SSC	27	19	27	17	29	15	14	18	20
		IR	47	52	20	43	25	17	22	56	35

### Comparative Genomic Analysis in the Nine *Musa* Chloroplast Genomes

**FIGURE 4:**
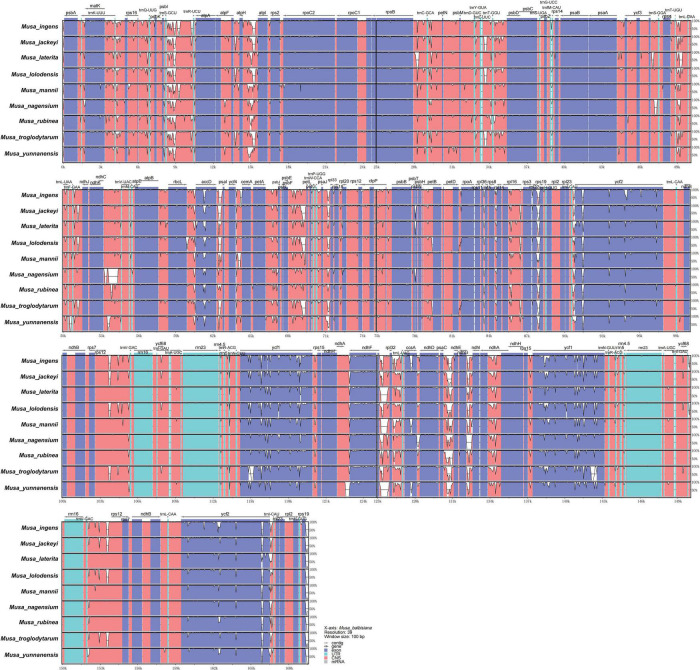
mVISTA map of chloroplast genome of nine species of *Musa*. Sequence identity plot comparing the chloroplast genome of nine *Musa* species. The vertical scale indicates the percentage of identity, ranging from 50 to 100%. The horizontal axis indicates the coordinates within the chloroplast genome. Genome regions are color-coded as protein-coding, rRNA, tRNA, intron, and conserved non-coding sequences (CNS).

**FIGURE 5:**
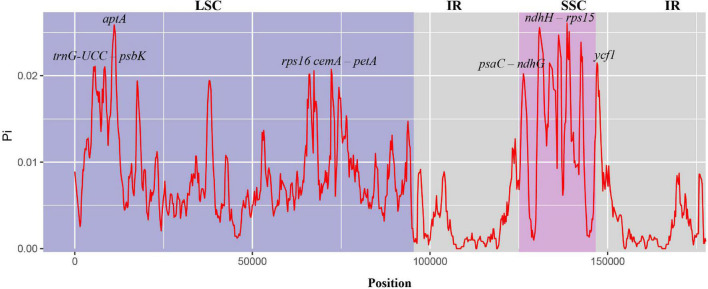
Nucleotide diversity in chloroplast genomes of nine species of *Musa*. The abscissa represents the position, and the red line represents the average of the nucleotide variations of the nine species (Pi).

To study the level of sequence polymorphism, we used DnaSP6 and mVISTA programs to calculate the genetic differences between nine *Musa* plants and compared the whole chloroplast genomes (Figure 4) with reference sequence of *M. balbisiana* set. In this study, the IR region variation of the *Musa* chloroplast genome was lower than that of LSC and SSC. In the coding region, the *ycf1*, *accD*, and *ycf2* of were quite different from each other. In general, non-coding regions were more susceptible to mutations than coding regions. Chloroplast genome of *Musa* is also consistent with this characteristic, and high variable regions are mainly found in IGS, such as *psbI–atpA, atpH–atpI, rpoB–petN, psbM–psbD*, *ndhF–rpl32, psaC–ndhE*, and *ndhG-ndhI.* These hot spots can be applied to DNA barcode encoding and phylogenetic analysis of *Musa* genus. With the rapid development of the chloroplast genome, comparing the differences in chloroplast genome sequences of different taxa can, it not only effectively screen out information-rich DNA fragments, but also promote the development of species identification and population diversity. The nucleotide variation (Pi) of nine species ranged from 0 to 0.08264, with an average value of 0.00792 (Supplementary Table 7). The average Pi of the SSC area was 0.01188, the average Pi of the LSC area was 0.00862, and the average Pi of the IR area was 0.00502. It can be seen that inverted repeats were more conservative than the single copy regions, and the coding regions were more conservative than the non-coding regions (Figure 5).

### Phylogenetic Analyses

**FIGURE 6:**
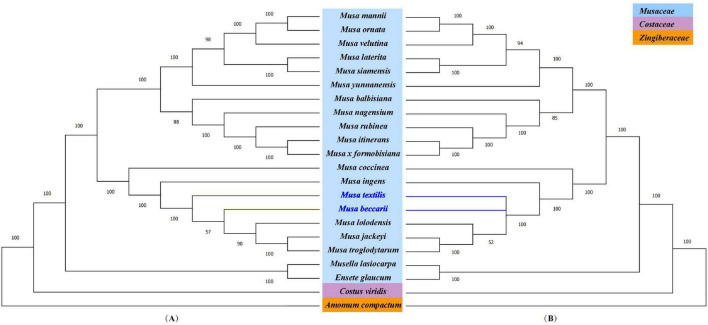
Phylogenetic analysis. **(A)** Phylogenetic tree based on the complete chloroplast genome. **(B)** Phylogenetic tree based on shared protein-coding genes. *Costus viridis* and *Amomum compactum* were selected as out groups. Numbers at branch nodes are bootstrap values.

To further understand the phylogenetic status of *Musa* plants and their relationship with other closely related species, the chloroplast whole genome and the shared protein-coding genes of 22 *Zingiberales* plants (including 18 *Musa* species) were used to constructed phylogenetic tree using maximum-likelihood (ML) method and bootstrap with 1,000 times iteration (Figure 6). The 22 *Zingiberales* plants were clustered as a large group, including many important crops, such as abaca (*M. textilis*), an excellent raw material for making specialty paper, and the primary sources of high-quality fiber-Abacá and Chinese dwarf banana, which was regarded as an essential Chinese medicinal material and rare ornamental plant, etc. The bootstrap values for almost all relationships inferred from all chloroplast genome data were very high. The results of the evolutionary tree we constructed can be divided into approximately four parts, which are *M. mannii*–*M. yunnanensis*, *M. balbisiana*–*Musa formobisiana*, *Musa coccinea*–*M. troglodytarum* and outgroups. In third part of the two evolutionary trees, there is a slight divergence, the Figure 6A showed that *M. textilis* and the sub-branches containing *M. beccarii*, *M. lolodensis*, *M. jackeyi*, and *M. troglodytarum* had sister relationship, and Figure 6B indicated that *M. textilis* and *M. beccarii* were sister species. *Musa lasiocarpa* is closer to *Ensete glaucum* of outgroups than to othe*r* 17 *Musa* species. The phylogenetic tree of nine *Musa* plants showed that *Musa* L. sect. *Musa* and *Musa* sect. *Callimusa* had a sister relationship, which was further verifying the latest *Musa* species classification (Weiner et al., 2019).

## Discussion

### Comparison of Chloroplast Genomes in the *Musa* Species

The chloroplast genome of angiosperms has made essential contributions to the study of phylogeny and the analysis of evolutionary relationships in phylogeny (Lee et al., 2019). The rich information in the chloroplast genome is very suitable as a DNA barcoding for species identification (Millen et al., 2001). However, among the 86 species belonging to *Musa* genus, there was very little analysis of complete chloroplast genomes. At this stage, only the complete chloroplast genomes of few species have been reported (Martin et al., 2013; Shetty et al., 2016; Liu et al., 2018; Feng et al., 2020), herein, we have added nine *Musa* species. The chloroplast genomes of most land plants are highly conserved, while during the evolution of angiosperms, one of the most fluid chloroplast genes, *infA*, was discovered (Millen et al., 2001). The *chlB*, *chlL*, *accD*, *ycf1*, *ycf68*, *infA*, *ycf15*, *ycf2*, *rpl22*, *rps16*, *rpl23*, *ndhF*, *chlN*, and *trnP* (GGG) genes in the plastid genome of some angiosperms were observed to be missing (Liaud et al., 1990; Liu and Xue, 2005; Jansen et al., 2007; Sheng et al., 2021). Among them, the deletion of four genes [*chlB*, *chlL*, *chlN* and *trnP* (GGG)] represents the homomorphism of flowering plants (Shahzadi et al., 2020). The deletions of the above four genes were found in the chloroplast genomes of all nine *Musa* species, including the missing of *ycf15* and *ycf68*. *M. laterita*, *M. mannii*, *M. nagensium*, *M. rubinea*, and *M. yunnanensis* all had two *rps19* genes, but only one in the chloroplast genomes of the other four species. This phenomenon is consistent with the classification of previous studies that the first five *Musa* sps. belong to *Musa* L. sect. *Musa*, and the last four species belong to *Musa* sect. *Callimusa* (Jiang et al., 2017).

Codon usage bias helped revealing the interaction between the chloroplast genome and its nuclear genome (Yang Y. et al., 2018). In many previous studies, the codons for leucine and isoleucine are the most common codons in the chloroplast, and the codons for cysteine are the least (Asaf et al., 2017b; Yang Y. et al., 2018; Shahzadi et al., 2020). The nine *Musa* species in this study also meet this feature. In the chloroplast genome of angiosperms, most codons showed higher A/T preference in the third codon. Our results followed this trend, and this phenomenon was also observed in *Forsythia suspensa* (Tian et al., 2018), *Epipremnum aureum* (Abdullah et al., 2020), *Zingibereae* sp. (Saina et al., 2018a), two Artemisia species (Piot et al., 2018), and other species. The main reason for this situation may be related to the abundance of A or T in the IR region (Chen et al., 2015).

Long repeats (LR) were essential when analyzing genome reorganization, rearrangement, and phylogeny, or inducing substitutions and insertions in the chloroplast genome (Chumley et al., 2006). We detected 86–324 LRs in nine *Musa* species, most of which were located in the IR region. This phenomenon was different from some species (Tian et al., 2018; Abdullah et al., 2020; Zhu et al., 2021). The IR regions of *Musa* sp. stabilizes plastid chromosomes through a repair mechanism induced by homologous recombination (Maréchal and Brisson, 2010). At the same time, our analysis shows that the proportion of LRs of *M. laterita*, *M. mannii*, *M. nagensium*, *M. rubinea*, and *M. yunnanensis* in the IR regions were greater than that of the other four species, which also may play a role in the genetic diversity and evolution of different *Musa* branches.

In the chloroplast genome, SSR was considered an important role in population genetics and phylogenetic analysis (Terrab et al., 2006). The number of SSRs were detected in the nine *Musa* species ranged from 73 to 93. The distribution of SSRs in the LSC region was higher than that in the IR and SSC region. At the same time, analysis shows that SSRs were mainly distributed in non-coding regions. These results were supported by previous studies on the chloroplast genome of angiosperms (Kim et al., 2009; Xu et al., 2012; Cheng et al., 2017). The SSRs analysis in this study showed that single nucleotide SSRs (A/T) had the highest content among the nine *Musa* plants, reaching 334, and mono-/di-/tri-/tetra-nucleotide repeats accounted for 86.17–94.52%, the penta- or hexanucleotide repeats were very few. The AT content in the chloroplast genome of nine *Musa* plants were higher than the GC content, and SSRs shows a strong AT bias, which was a common phenomenon in the chloroplast genome of higher plants (Kuang et al., 2011; Lei et al., 2016). Repetitive sequences played a vital role in generating insertion mutations and substitution mutations (McDonald et al., 2011). Previous studies have shown widespread substitutions and deletions in the LSC and SSC regions of the chloroplast genome (Ahmed et al., 2012).

### Comparison of the Sequences Within *Musa* Complex Species and Phylogenetic Relationships

The IR/LSC boundary position was not fixed during the evolution of angiosperms but can expand and contract moderately (Ahmed et al., 2012). The large inverted repeat sequence may be directly related to the structural conservation of the chloroplast genome (Palmer and Thompson, 1982). In some angiosperms, the expansion or contraction of IR is usually accompanied by the change of gene position. For example, the *ycf1* gene often is pseudogene because it crosses the boundary between LSC–IR and SSC–IR (Saina et al., 2018b; Yang Z. et al., 2018; Shahzadi et al., 2020). In our research, we divided these nine species into three categories based on the location of *rps19* gene. In contrast, the *M. yunnanensis* in the third category and the four species in the first category belong to *Musa* L. sect. *Musa*, the three species in the second category and the *M. ingens* in the third category all belong to *Musa* sect. *Callimusa*. In the chloroplast genome of *M. yunnanensis*, we speculated that two *rps19* genes appear in the LSC region due to the contraction of the IR region. In contrast, the *ndhB* gene remained in the IR region, thus evolving into a part of *Musa* sect. *Callimusa*. The shrinkage or expansion of the IR region was one of the essential features for understanding the evolution and structure of the chloroplast genome (Jiang et al., 2017).

The SNP distributions of the nine species were very similar. The SNPs of each species account for the highest proportion in the LSC region. Except for *M. nagensium*, the SNPs of the other species in the IR region were more than the SSC region. We also analyzed the insertions and deletions of the chloroplast genomes of nine species, and the results found that they have similar rules as SNPs. In that case, it is possible to predict mutation hot spots and better study population genetics and analyze the phylogenetic relationship of species (Du et al., 2017; Keller et al., 2017).

*K*_*a*_/*K*_*s*_ is used to assess nucleotides’ natural selection pressure and evolution rate, which is a meaningful marker in species evolution (Li et al., 1985). In our study, *K*_*s*_ was much higher than *K*_*a*_, which means that the evolution of *Musa* species was relatively slow. Only two genes (*ycf1* and *ycf2*) were under positive selection, and this was also somewhat different from the species of the Zingiberaceae (Liang and Chen, 2021). Consistent with many previous studies, the evolution of photosynthesis genes was slower than other types of protein-coding genes (Wicke et al., 2011; Saina et al., 2018a; Tian et al., 2018). Genes under positive selection often inserted many repetitive amino acid sequences to varying degrees, which may be evidence of adaptation to new ecological conditions or the result of co-evolution (Piot et al., 2018).

The chloroplast genome sequence contains highly variable regions. Finding more regions with a higher evolution rate is helpful to distinguish closely related species or genus, which is of great significance to the study of DNA barcodes (Dong et al., 2012). The chloroplast genomes contained two huge genes, *ycf1* and *ycf2*, which were indispensable chloroplast genes in higher plants (Drescher et al., 2000). The proteins that control transcription play an important role in cell survival. In the chloroplast genomes of most flowering plants, the *accD* gene encodes the β-carboxyl transferase subunit of acetyl-CoA carboxylase, which is essential for plant leaf development (Kode et al., 2005). Since they are all protein-coding genes, they may provide information about the evolution of *Musa* plants. Our comparative analysis identified several non-coding sites (*psbI*–*atpA, atpH*–*atpI, rpoB*–*petN, psbM*–*psbD, ndhf-rpl32, psaC-ndhE*, and *ndhG-ndhI*) and three genes (*ycf1, ycf2*, and *accD*). These mutation hotpots with high nucleotide diversity were particularly suitable for *Musa* genus’ further molecular phylogeny and population genetics research.

In recent years, many studies have used protein-coding regions or chloroplast whole-genome sequence for phylogenetic analysis (Henriquez et al., 2014). The results of this study revealed the genetic relationship between *Musa* plants. It is generally believed that *Musa* genus includes *Musa* sect. *Rhodochlamys*, *Musa* sect. *Eumusa*, *Musa* sect. *Australimusa*, and *Musa* sect. *Ingentimusa* (Cheesman, 1947). Currently, the *Musa* genus is divided into two sections, *Musa* L. sect. *Musa* and *Musa* sect. *Callimusa* (Häkkinen, 2013). At the same time, the 19 unlinked nuclear genes confirmed the close relationship of *Australimusa* and *Callimusa* sections and showed that *Eumusa* and *Rhodochlamys* sections are not reciprocally monophyletic (Christelov et al., 2011). Our analysis revealed that *Musa* sect. *Rhodochlamys* and *Musa* sect. *Eumusa* were sisterly related to *Musa* sect. *Australimusa* and *Musa* sect. *Ingentimusa*. This result further verified that *Musa* L. sect. *Musa* included *Musa* sect. *Rhodochlamys* and *Musa* sect. *Eumusa*, and *Musa* sect. *Callimusa* comprised *Musa* sect. *Australimusa* and *Musa* sect. *Ingentimusa*. Based on the evolutionary tree, we also found that *M. lasiocarpa* is a basal species in the genus of *Musa* (Novák et al., 2014), which will help to deduce the time of origin of *Musa*. In addition, the results we obtained were different from previous studies (Liu et al., 2018; Feng et al., 2020). For example, the findings of their results concluded that *M. textilis* was the sister group of *M. balbisiana* and *M. beccarii* was closer to the roots of the evolutionary tree than *Musa itinerans*, which may be related to the other genomic regions and species collected. At present, the phylogenetic analysis of *Musa* species we have done was based on the complete chloroplast genome and protein-coding genes were the most comprehensive, which provided a theoretical foundation and technical support for the development and utilization of *Musa* plants resources.

## Conclusion

In this study, we reported and compared the complete chloroplast genomes of nine *Musa* species in the first time, greatly increasing the available molecular sequences for this genus. The complete chloroplast genomes of these nine species were typical circular double-stranded quadripartite structure and ranged from 167,975 to 172,653 bp in the length. We analyzed the sequences of the chloroplast genomes of nine *Musa* species, such as the sequence length of each region, the number of different types of genes, and the types of intron genes. Codon bias analysis presented an extensively preferences for codons containing A/T at the 3′ end, especially for those who showed RSCU greater than one. We detected most of repetitive sequence existed in range of 30–50 bp. As shown in *K*_*a*_/*K*_*s*_ evaluation, 77 of protein-coding genes was relatively stable during evolution process, while two genes (*ycf1* and *ycf2*) were under positive selection. Our research also revealed that all nine *Musa* species have *ndhA* genes that spanned the SSC and IRa regions, and notably, the *rps19* gene was entirely located in the IRb regions, suggesting that this phenomenon may occur with the contraction IRb area. SNP and InDels analysis divided nine *Musa* species into two groups in terms of the abundance and distribution of nucleotide polymorphic phenomenon, which was further confirmed by the phylogenetic tree. In summary, comparing the chloroplast genomes of *Musa* can deepen our understanding of the evolution of the Musaceae and may be suitable for the phylogenetic analysis and classification of *Mus*a genus.

## Data Availability Statement

The original contributions presented in the study are publicly available. The data that support the findings of this study are openly available in the Genbank database at https://www.ncbi.nlm.nih.gov/ under accession number NC_056826 - NC_056834.

## Author Contributions

CJ and CS: conceptualization. HC, XW, and SW: data curation. SW: formal analysis. CS and SW: funding acquisition. CJ, HC, and XW: investigation. WS: methodology. WS, CS, and SW: project administration. WS and ZC: software, visualization, and writing – review and editing. CS: supervision. HC and XW: validation. WS and CJ: writing – original draft. All authors contributed to the article and approved the submitted version.

## Conflict of Interest

The authors declare that the research was conducted in the absence of any commercial or financial relationships that could be construed as a potential conflict of interest.

## Publisher’s Note

All claims expressed in this article are solely those of the authors and do not necessarily represent those of their affiliated organizations, or those of the publisher, the editors and the reviewers. Any product that may be evaluated in this article, or claim that may be made by its manufacturer, is not guaranteed or endorsed by the publisher.

## Supplementary Material

Supplementary Table 1 Intron details.

Supplementary Table 2 Codon usage details all nine *Musa* species.

Supplementary Table 3 *K*_*a*_/*K*_*s*_ raw data all nine *Musa* species.

Supplementary Table 4 RNA editing sites raw data of all nine *Musa* species.

Supplementary Table 5 Detail of SSR and long repeats.

Supplementary Table 6 SNP and InDels statistics.

Supplementary Table 7 Raw data of Pi values.

Supplementary Table 8 Information of species in phylogenetic tree.
